# Impact of resilience enhancing programs on youth surviving the Beslan school siege

**DOI:** 10.1186/1753-2000-4-11

**Published:** 2010-04-22

**Authors:** Stefan Vetter, Igor Dulaev, Mario Mueller, Robert R Henley, William T Gallo, Zalina Kanukova

**Affiliations:** 1 Centre for Disaster and Military Psychiatry, University of Zurich, Switzerland; 2 North Ossetian Institute of Humanitarian and Social Research, Vladikavkaz, Russian Federation; 3 CUNY School of Public Health and Brookdale Center for Healthy Aging and Longevity, Hunter College, USA

## Abstract

The objective of this study was to evaluate a resilience-enhancing program for youth (mean age = 13.32 years) from Beslan, North Ossetia, in the Russian Federation. The program, offered in the summer of 2006, combined recreation, sport, and psychosocial rehabilitation activities for 94 participants, 46 of who were taken hostage in the 2004 school tragedy and experienced those events first hand. Self-reported resilience, as measured by the CD-RISC, was compared within subjects at the study baseline and at two follow-up assessments: immediately after the program and 6 months later. We also compared changes in resilience levels across groups that differed in their traumatic experiences. The results indicate a significant intra-participant mean increase in resilience at both follow-up assessments, and greater self-reported improvements in resilience processes for participants who experienced more trauma events.

## Introduction

In early September 2004, approximately 32 terrorists attacked a school in Beslan, North Ossetia, in the Russian Federation. At the time of the attack, the school was holding festivities to mark the first day of classes after summer holiday. Over 1300 adults and children were taken hostage in the siege, and during a three-day period of detention, 344 hostages died (including 186 children) and more than 700 were injured. This event had an extensive negative impact on the health of children, families, and the entire Beslan community [1].

For three years following the Beslan school hostage tragedy, the Swiss Department of Development and Cooperation (SDC) fully financed a number of psychosocial programs under the auspices of the North Ossetian Ministry of Education. These programs were offered by staff of the local psychosocial education centre "Doverie". From the perspective of resilience [2-5], a number of recreation, sport, and psychosocial rehabilitation activities were offered to support the recovery of youth in the Beslan area. One of these programs was a "resilience-enhancing program", which was set in the nearby mountain resort of Tsey in the summer of 2006. In this program, "resilience enhancement" was attempted by offering a variety of outdoor experiences under the guidance of caring, non-family adults who instructed participants in useful problem-solving strategies, as well as life and coping skills, in a community experience with supportive peers.

Resilience enhancement and development were selected as the focus of the program for two primary reasons. First, it was hypothesized that the youth involved in these programs would benefit from enhancement of their emotional, mental and social capacities to overcome the adversities they faced. Research has shown that building resilience among survivors of high-risk environments can help develop and/or maintain: social competence, empathy, caring, problem-solving skills, critical and creative thinking, task mastery, and a sense of purpose and social connectedness [6]. Problem-solving skills are a particularly strong predictor of improved resilience in children and youth in the long-term, as improved problem-solving skills can enhance the possibility that future life challenges will be resolved successfully [5,7-10]. And second, it was determined by the government that to intervene directly on trauma issues might produce alternative outcomes that would have regressive and destructive psychological affects on the children. Thus, the program needed to focus on strengths and capacity building.

This research project is based on the developmental psychology perspective of resilience, first conceptualized by Luthar, and later extended and applied by others [2,4,11-13]. Luthar's basic definition of resilience is: "A dynamic process encompassing positive adaptation within the context of significant adversity. Implicit within this notion are two critical conditions: 1.) An exposure to significant threat or severe adversity, and 2.) the achievement of positive adaptation despite major assaults on the developmental process" [2,3,14]. As defined by Luthar, resilience is neither a "trait" or "state," but rather a multi-dimensional dynamic process, which implies that it can occur in a person in different ways, at different times, in response to different situations [2,14]. Resilience thus exists as a potential that may be fostered and supported within individuals, without regard to age or developmental phase.

The importance of the Beslan program is primarily in its relevance to resilience-enhancing interventions in community-based settings - interventions that may be especially influential when undertaken during significant developmental transitions, such as entry into school, advancing to higher levels of school, moving from childhood into adolescence, from school into the workforce, and from youth into adulthood [2,3,14]. Thus, the results of effective resilience-fostering interventions can offer practitioners in the field unique opportunities for promoting positive adaptation in a variety of adverse situations, and assisting transitions through various developmental stages [3,5,14].

Past research on psychological resilience has identified four key protective factors [10,15-19] that can contribute to the development, support and sustaining of resilience processes in youth. They are: (a) healthy attachments to related and unrelated older adults, who provide them with support, encouragement, and guidance; (b) healthy and connected peer relationships; (c) effective problem-solving skills and coping strategies; and (d) community involvement, in support of the common good. These protective factors are interactive with resilience factors, as these both help develop and can later help sustain resilience processes and trajectories [5,16].

In Beslan, the goal was to examine a program that incorporated these four vital protective factors for enhancing the development of resilience processes. The approach was to combine sport activities, safety training, and various therapeutic rehabilitation activities in a group setting, where participants camped together away from their homes. Specifically, the program provided: (1) first aid, cardio-pulmonary reanimation (CPR), and life-saving rescue techniques; (2) mountaineering and survival skills training; (3) alpine sport activities, such as safe skiing, climbing, and alpine walking; and (4) informal arts, play, and supportive talk therapy sessions. It was anticipated that each of the components would foster resilience via multiple mechanisms. For example, the physical activities in the mountain wilderness were expected to build internal resilience though boosting confidence, supporting the experience of positive emotions, improving somatic health, and encourage a climate of solidarity, both with peer participants and adult leaders.

Further explication of the therapeutic component of the program may be useful. This element was primarily designed to offer participants assistance in sorting out emotions provoked by the activities involved in the other parts of the program. In the evenings, children played parlor games, sang songs, told stories, created art, and engaged in other similar leisure activities, all of which were facilitated by psychosocial therapists. These sessions were informal and primarily supportive in focus, with no special training offered to the therapists and no particular therapeutic approach or intervention emphasized. Therapy was administered as needed. That is, in cases where a child's behavior suggested that the daytime or evening activities provoked exacerbation of psychopathology, psychologists addressed the children's experiences and emotions within the group. In the rare case where children were extremely disruptive, a psychologist would remove them, and address the behavior or emotions in a one-to-one setting.

It is important to note that the actual outdoor activities and training were conducted by rescuers (emergency medical technicians and professional mountain guides) from the North Ossetian Search and Rescue Services, a department of the Russian Federal Ministry for Emergencies. All participating rescuers were professionals who had been involved in the liberation mission of the Beslan hostages, and were thus considered quite positively by the camp's participants. Additionally, all these programs were supported by staff of the local psychosocial education centre "Doverie". All psychologists were university qualified in the Russian Federation, and all attended specific courses for psycho-traumatology in Israel prior to participating in the camp.

The primary research objective of this project was to assess whether a youth's involvement in the overall program would result in enhanced resilience processes and towards more resilient life trajectories. In the absence of a comparison group, within-subject resilience was compared at three time points: before the program, at the completion of the program, and at a six-month follow-up assessment. A secondary objective was to investigate variations in the effect of the interventions across groups, which differed in their experiences of the 2004 Beslan school tragedy. For this study, the Connor-Davidson Resilience Scale (CD-RISC) [6], was utilized to measure resilience. At the time of the study, the CD-RISC was one of the few instruments in existence that expressly assessed resilience processes. The CD-RISC has been validated by a number of studies, and is also one of the few resilience instruments that have been used cross-culturally [20-24].

## Methods

### Data Collection and Sample

Between June 2006 and September 2006, eight consecutive resilience-enhancement program camps, with duration of one week each, were offered to 120 children of Beslan. An average of fifteen youth took part in each of the eight camps. Participants included hostages and non-hostages, both boys and girls. We used three means of recruitment for our program. First, we placed an announcement on a placard in Beslan's parish hall. Second, we enlisted a representative of the Ministry of Education to inform all school psychologists in the city of Beslan of our program. And third, we asked the staff at Beslan's Psychosocial Center to inform parents of children attending activities there. Although participation was open to all interested youth of Beslan who were age 10 and older, a majority were children who also took part in some activity of Beslan's Psychosocial Center. Of the children who did participate, the mean age at baseline was 13.32 years (SD = 1.39) with a participant range from age 10 to age 16. Our study sample is thus a convenience sample, not a representative one.

Ninety-eight of the 120 Beslan youth who participated in the programs responded to all of our surveys, whose administrations took place at three time points: (1) on the day of departure to the camp (baseline), (2) at the end of the program (follow-up 1), and (3) six months after the end of the program (follow-up 2). A research psychologist of the North Ossetian Institute of Humanitarian and Social Research conducted the assessments. The parents of the children were invited to be present during the assessments, but parental attendance was not mandatory. Data collection took place between June 2006 and March 2007. At the second follow-up, 4 children could not be reached for re-assessment. The final sample for analysis was therefore 94 youth. Forty-six of the sample members were hostages during the Beslan school crisis; the remaining 48 were non-hostages.

### Informed Consent & Ethics Clearance

Signed informed Consent letters from the families of all participating youths were obtained, stating that they understood the research and approved of their children's involvement in this study. Additionally, this study was given Institutional Review Board clearance and approval by the Beslan Authorities and by the University of Zurich.

### Measures

#### Outcome Variable

Resilience was measured with the Connor-Davidson Resilience Scale (CD-RISC), one of the few scales created to exclusively measure resilience [6,25,26]. The CD-RISC consists of 25 statements with 5 Likert-scaled responses. Responses are 0 ("not true at all"), 1 ("rarely true"), 2 ("sometimes true"), 3 ("often true"), and 4 ("true nearly all the time"). The potential range of the CD-RISC is thus 0 - 100, with higher values reflecting greater resilience. The CD-RISC has been found to be both valid and reliable, with good internal consistency [24,25,27]. Other evidence suggests that the CD-RISC has significant convergent validity with other tests that measure aspects of resilience, including the "Kobasa Hardiness Scale" [28], "Perceived Stress Scale" [29], "Stress Vulnerability Scale" [30], "Sheehan Disability Scale" [31] and the "Sheehan Social Support Scale" [30]. Positive correlations have also been demonstrated with the "Rosenberg Self-Esteem Scale" [32], the "Life Satisfaction Scale" [33], and the "NEO 5 Factor Inventory" [34].

The authors of the CD-RISC have asserted its utility in measuring the effectiveness of contemporary resilience interventions and exploring resilience qualities within individuals. The primary motivation for using the CD-RISC was that it permits assessment of whether the modification of strengths and positive attributes may lead to diminishing problems among individuals who engage in adaptive pursuits [25,27]. We used a recently released, Russian-language version of the CD-RISC, which was translated and back translated by specialists who were authorized by the authors of the original instrument. Respecting local requests to monitor only resilience, posttraumatic stress disorder (PTSD) was not assessed, accepting the limitations emerging from such a renouncement. As an alternative, a basic demographic assessment regarding self-reports of direct and indirect traumatic experiences of the hostage tragedy was added.

#### Explanatory variables

Age was a continuous variable. Gender was coded as 1 = male, 0 = female. Four measures captured the extent of traumatic experience of the 2004 Beslan school tragedy: One variable was a binary measure ("hostage status") that indicated whether a program participant was taken hostage in 2004 or not (1 = hostage, 0 = non-hostage). The second variable ("losses") was based on the total number of deaths that participants experienced, which included the sum of the number of relatives, friends, and teachers who died in the school incident. The third variable ("injuries") captured direct harm experienced by hostage participants during the school siege, or for non-hostages, direct harm to someone that they knew. The fourth variable was a summary measure of the second and third variables; it therefore combined the total number of losses and injuries. To create the losses and injuries measures used in our analyses, the first step was to separately tally the number of losses and injuries at the participant level. We then created three categorical variables, each of which had 3 levels that were coded as: [losses] 0 losses (referent), 1 loss, and 2+ losses; [injuries] 0 injuries (referent), 1-2 injuries, and 3+ injuries; [summary] 0 losses/injuries (referent), 1-2 losses/injuries, and 3-7 losses/injuries.

### Statistical analysis

We had four objectives for the data analysis: (1) to describe the sample; (2) to compare cross-sectional resilience scores, at baseline and follow-up assessments, by age, gender, and hostage status; (3) to analyze the effect of the intervention on within-participant changes in CD-RISC scores, and to explore variation in the intervention effect by the extent of reported direct and indirect trauma experiences from the school tragedy; and (4) to test the reliability of the CD-RISC in our sample.

We described the sample (Objective 1) with means and standard deviations (continuous variables) or frequency and percent (categorical variables). Objective 2 was accomplished with simple Pearson-correlations, for analyzing the cross-sectional association between CD-RISC and age, and ANOVAs, for cross-sectional estimating of differences in CD-RISC scores by gender and hostage status, at baseline and follow-up points. Post-hoc analyses (Bonferroni tests) were performed to determine statistical significance in all cross-sectional mean differences. We used repeated measures analysis (Generalized Estimating Equations) to test for changes in mean resilience over time (Objective 3). We estimated two models: The first model assessed only within-subject time effects, and the second model additionally considered the between-subject traumatic experience variables. Four specifications of the second model were fitted: one in which time was interacted with hostage status; one in which time was interacted with the categorical measure of losses; one in which time was interacted with the categorical measure of injuries; and a final specification in which time was interacted with the categorical summary measure of losses and injuries. Effect sizes (Cohen's d) [35] were calculated to indicate strength in mean CD-RISC differences between baseline and follow-up periods [36]. For Objective 4, we calculated Pearson correlations between measurement points to assess CD-RISC test-retest reliability. Internal consistencies were evaluated by Cronbach's Alpha. The data analyses were performed with SPSS statistical package (Version 15.0) and SAS Version 9.1.

## Results

### Description of Sample

**Figure 1:**
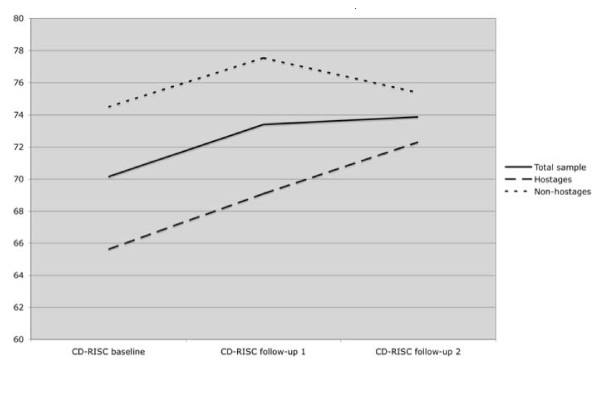
**CD-RISK scores over time**.

The total sample consisted of 94 youth, of which 39.4% were female and 60.6% were male. The mean age at baseline was 13.32 years (SD = 1.39) with a range of 10 to 16. Mean scores of the CD-RISC for the total sample were: 70.15 (SD = 14.06) at baseline, 73.40 (SD = 12.60) at follow-up 1, and 73.87 (SD = 11.58) at follow-up 2 (see Figure 1).

Fifty-five participants (58.5%) did not report experiencing any losses (i.e., deaths), and this group included 14 hostages and 41 non-hostages. Twenty-two participants (23.4%; 15 hostages/7 non-hostages) lost one person, ten participants (10.2%; only hostages) lost two persons, and seven participants (7.4%; only hostages) lost three persons during the school shooting incident. Regarding injuries, 30 participants (31.9%; 2 hostages/28 non-hostages) were neither injured nor knew any other injured persons, while 35 participants (37.2%; 15 hostages/20 non-hostages) reported one injury or knowing an injured person, 12 participants (12.8%; only hostages) reported having two injuries or knowing others who had, and 13 participants (13.8%; only hostages) indicated having three injuries or knowing others with three injuries. Additionally, two participants reported having experienced four injuries (2.1%; only hostages), and two participants experienced five injuries in the events (2.1%; only hostages). Twenty-one non-hostages (43.8%) and one hostage (2.2%) did not suffer any type of injury or loss. (See Table 1 for further details.)

**Table 1:** Potential traumatizing events from Beslan hostage

	Full Sample Frequency (%) (N = 94)	Hostages Frequency (%) (N = 46)	Non-Hostages Frequency (%) (N = 48)
**LOSSES**			
Loss of mother/father	2 (2.1%)	2 (4.3%)	---
Loss of brother/sister	4 (4.3%)	4 (8.7%)	---
Loss of other relatives	15 (16.0%)	13 (28.3%)	2 (4.2%)
Loss of friends	29 (30.9%)	24 (52.2%)	5 (10.4%)
Loss of teacher	13 (13.8%)	13 (28.3%)	---
*Losses total*	63	56	7

**INJURIES**			
Injured themselves	11 (11.7%)	11 (23.9%)	---
Injury of mother/father	3 (3.2%)	3 (6.5%)	---
Injury of brother/sister	6 (6.4%)	6 (13.0%)	---
Injury of other relatives	13 (13.8%)	8 (17.4%)	5 (10.4%)
Injury of friends	58 (61.7%)	43 (93.5%)	15 (31.3%)
Injury of teacher	25 (26.6%)	25 (54.3%)	---
*Injuries total*	116	96	20

**Total number of losses and injuries**	179	152	27

### Cross-sectional CD-RISC scores by demographic variables and hostage status

No significant gender differences were found at any of the three time points (diffs = 3.21-4.21; ts = 1.21-1.72; df = 92; n.s.). Similarly, age was not associated with the CD-RISC at any time (r = .00 - .17; n.s.). Consequently, subsequent analyses were performed without controlling for age and gender.

Significant mean differences in CD-RISC scores by hostage status were found at baseline (diff = 8.89; t = 3.21; df = 92; p = .002) and follow-up 1 (diff = 8.45; t = 3.44; df = 92; p = .001). Mean baseline CD-RISC mean scores were 65.61 (SD 16.09) for hostages and 74.50 (SD 10.19) for non-hostages. At follow-up 1, CD-RISC mean scores were 69.09 (SD 14.36) for hostages and 77.54 (SD 8.99) for non-hostages. Significant differences in CD-RISC were not detected at follow-up 2 (diff = 3.07; t = 1.29; df = 92; n.s.). Mean scores of 72.30 (SD 13.60) for hostages and 75.38 (SD 9.14) for non-hostages were calculated. Figure 1 displays a graphical presentation of the CD-RISC course over time for the total sample as well as stratified by hostage status.

### Impact of the intervention (time) and differences by traumatic experience

The results of the repeated measures analysis (Table 2) indicate a significant time effect on changes in CD-RISC scores for the overall sample, which suggests a measurable impact of the resilience-building intervention. The findings in the full sample (Model 1) suggest that the average participant in the resilience-building camp had a significant increase in CD-RISC resilience scores from baseline to follow-up 1 (p < .001) and from baseline to follow-up 2 (p < .001).

**Table 2:** Generalized Estimating Equations results on time and time interaction with traumatic experiences

	Model 1: Time Only	Model 2: Specification 1 Hostage Status Interaction	Model 2: Specification 2 Losses Interaction	Model 2: Specification 3 Injuries Interaction	Model 2: Specification 4 Losses/Injuries Interaction
***Main effects***					
*Effect of Time*					
Follow-up 2	3.72 (.84)***	6.69 (1.34)***	2.25 (.89)*	2.30 (.91)*	3.23 (1.11)***
Follow-up 1	3.25 (.38)***	3.48 (.58)***	3.22 (.49)***	3.60 (.61)***	4.00 (.78)***
Study baseline	*Ref*.	*Ref*.	*Ref*.	*Ref*.	*Ref*.

***Effect of Traumatization***					
*Hostage Status*					
Hostage		-8.89 (2.76)***			
Non-hostage		*Ref*.			
*Losses*					
2+ losses			-13.38 (3.93)***		
1 loss			-2.70 (3.51)		
No losses			*Ref*.		
*Injuries*					
3+ injuries				-11.67 (3.92)**	
1-2 injuries				-1.41 (2.83)	
No injuries				*Ref*.	
*Losses/Injuries*					
3+ losses/injuries					-11.78 (3.51)***
1-2 losses/injuries					-0.64 (2.95)
No injuries					*Ref*.

***Interactions of time with*** ***traumatization***					
*Hostage Status*					
Follow-up 2 * hostage		5.82 (1.58)***			
Follow-up 1 * hostage		0.44 (0.76)			
Study baseline * hostage		*Ref*.			
*Losses*					
Follow-up 2 * 2+ losses			6.69 (2.44)**		
Follow-up 2 * 1 loss			1.11 (2.10)		
Follow-up 2 * 0 losses			*Ref*.		
Follow-up 1 * 2+ losses			1.25 (.93)		
Follow-up 1 * 1 loss			-.81 (.95)		
Follow-up 1 * 0 losses			*Ref*.		
*Injuries*					
Follow-up 2 * 3+ injuries				5.17 (2.06)*	
Follow-up 2 * 1-2 injuries				.98 (1.65)	
Follow-up 2 * 0 injuries				*Ref*.	
Follow-up 1 * 3+ injuries				-.42 (.86)	
Follow-up 1 * 1-2 injuries				-.53 (.87)	
Follow-up 1 * 0 injuries				*Ref*.	
*Losses/Injuries*					
Follow-up 2 * 3+ losses/injuries					4.99 (1.79)**
Follow-up 2 * 1-2 losses/injuries					0.07 (0.95)
Follow-up 2 * 0 losses/injuries					*Ref*.
Follow-up 1 * 3+ losses/injuries					-2.11 (1.70)
Follow-up 1 * 1-2 losses/injuries					-1.63 (0.98)
Follow-up 1 * 0 losses/injuries					*Ref*.
Intercept	70.15 (1.44)***	65.61 (2.35)***	73.20 (1.58)***	72.97 (1.85)***	73.95 (2.20)***

Note: Table values represent estimated coefficient (standard error). *** p < .001, **p < .01.

The results also indicate differences in mean CD-RISC change by traumatic experience. Across hostage status (Model 2, Specification 1), we find that, on average, hostages revealed a greater increase in CD-RISC scores than non-hostages at follow-up 2 (p < .001), although no differences were detected by hostage status at follow-up 1. Variation in CD-RISC score changes at follow-up 2 was also suggested across loss categories. In this case, participants who experienced two or more losses (Model 2, Specification 2) had greater gains to resilience between baseline and follow-up 2 than those who experienced no losses (p < .01). Similarly, participants who experienced three or more injuries (Model 2, Specification 3) had greater gains to resilience between baseline and follow-up 2 than those who experienced no injuries (p < .05). These findings are reflected in the analysis of the summary measure (Model 2, Specification 4). Participants who experienced more 3 or more losses/injuries had greater gains to resilience between baseline and follow-up 2 than those who experienced no losses/injuries (p < .01).

Effect sizes (d) for the relevant groups are presented in Table 3. The effect sizes from the start of the program to its end (i.e., to follow-up 1) were d = 0.23 for hostages and d = 0.32 for non-hostages. The equivalent effect sizes were d = .31 for participants with 2+ losses and .29 for those with no losses. For injuries, the effect sizes were d = .22 for participants with 3+ injuries and .37 for participants with no injuries. According to Cohen's criteria, the effect size for both groups of highly traumatized participants is small.

**Table 3:** Means of CD-RISC with effect sizes

	CD-RISC baseline	CD-RISC follow-up1	CD-RISC follow-up2	Effect size (d) baseline-follow-up 1	Effect size (d) baseline-follow-up 2
	M (SD)	M (SD)	M (SD)		
***Full Sample***					
Total (N = 94)	70.15 (14.00)	73.40 (12.60)	73.87 (11.54)	.24	.29

***Hostage Status***					
Hostages (N = 46)	65.61 (15.98)	69.09 (14.25)	72.30 (13.50)	.23	.45
Non-hostages (N = 48)	74.50 (10.12)	77.54 (8.92)	75.38 (9.07)	.32	.09

***Losses***					
2+ losses (N = 17)	59.82 (14.96)	64.29 (14.30)	68.76 (12.43)	.31	.65
1 loss (N = 22)	70.50 (14.86)	72.91 (13.09)	73.86 (13.60)	.17	.24
No losses (N = 55)	73.20 (11.76)	76.42 (10.23)	75.45 (9.85)	.29	.21

***Injuries***					
3+ injuries (N = 17)	61.29 (14.37)	64.47 (13.91)	68.76 (13.23)	.22	.54
1-2 injuries (N = 47)	71.55 (14.78)	74.62 (12.61)	74.83 (11.26)	.22	.25
No injuries (N = 30)	72.97 (10.21)	76.57 (8.97)	75.27 (10.20)	.37	.23

***Losses/Injuries***					
3+ losses/injuries (N = 28)	62.18 (14.74)	66.25 (14.14)	70.39 (12.61)	.28	.59
1-2 losses/injuries (N = 44)	73.32 (13.33)	75.68 (11.46)	74.43 (11.58)	.19	.09
No losses/injuries (N = 22)	73.95 (10.57)	77.95 (8.70)	77.18 (9.27)	.41	.32

The effect sizes associated with 6-month follow-up imply a different pattern. At follow-up 2, hostages' CD-RISC mean scores drew close to the baseline mean scores of non-hostages, and the effect size between baseline and 6-month follow-up therefore increased to d = 0.45, which suggests a medium effect of the overall program. On the other hand, the equivalent effect size for non-hostages was calculated to be d = 0.09, which suggests a negligible-to-null effect of the intervention at long-term follow-up. A similar relative change occurred with regard to the losses groups, with a stronger effect size (d = .65) emerging among the participants with 2+ losses, and a weaker effect (d = .21) appearing among the referent (no losses) group. Among the groups experiencing injuries, the relative changes were consistent, with a stronger effect size (d = .54) emerging among the participants with 3+ injuries, and weaker effect (d = .23) appearing among the referent (no injuries) group. The general pattern of effect sizes for the combined classes of injuries and losses mirrors those of the disaggregated categories, and thus warrants no further discussion.

### Reliability of used instrument

Cronbach's Alpha for the CD-RISC was .88 for all three measurement points. Test-retest reliability was assessed for CD-RISC assessments at the three time points. The association between baseline and follow-up 1 was .97 (p < .001); between follow-up 1 and follow-up 2 was .84 (p < .001); and .81 (p < .001) for the total period, indicating a high rank-order stability over time. Overall, the CD-RISC shows satisfactory-to-high reliabilities. In this sample, the CD-RISC shows high degrees of homogeneity at all measurement periods and high sensitivity to changes in resilience.

## Discussion

Since September 2004, when members of the Beslan community were held hostage by terrorists, merely one study [37] and one clinical report [38] have addressed the community's posttraumatic stress reaction. Two further articles have chronicled narratives of caregivers [39] and observations of the affected community [40]. The current study takes a new and different perspective, evaluating a resilience intervention program for Beslan youth offered during summer holidays of 2006. Adopting a resilience perspective, rather than a trauma and psychopathology perspective, the primary goal of this program was to promote physical, mental, and social well-being in youth of the Beslan community. Specifically, the program was designed to enhance the resilience of participants via acquisition of new skills and strategies, and social engagement with peers and older non-family adults. The program was undertaken with two major scientific objectives in mind: to evaluate intra-subject change in resilience at the end of the program, and then 6 months later; and to determine whether this self-reported resilience differed, cross-sectionally and in terms of change, according to the number of trauma experiences resulting from the Beslan school tragedy. In support of the second objective, we also sought to document the extent of injuries and death associated with program participants.

The first suggestion of our findings is that the program intervention had a measurable effect on subsequent self-reported resilience levels within individuals. The average sample member reported statistically significant increases in resilience from the baseline value, at both the end of the one-week intervention and at 6-month follow-up. The second relevant finding is that participants who had been taken hostage during the school tragedy differed from non-hostages in two ways: one, hostages had lower resilience scores than non-hostages at baseline, and immediately after the program; and two, hostages experienced greater gains in resilience than non-hostages six months after the program's completion. A similar finding was implied by the analysis of differential effects according to loss and injury experience. That is, participants in the highest losses and injuries categories had significantly lower resilience than those in the lowest categories at the baseline measure, but reported greater increases in resilience from the baseline to the second follow-up.

There are numerous limitations of note in this study. First and foremost, our intervention was performed without a control group, which weakens any argument for the efficacy of the resilience enhancing program. We recommend that future studies build upon our pilot findings by incorporating quasi-experimental elements. Previous literature on school-based interventions for non-environmental traumatic events suggests that the use of a wait-list control group provides a feasible method for analysis [41-43]. Nevertheless, as wait-list controls are generally not blinded to treatment status, this approach can results in biased results. An alternative to the wait-list control approach is the use of a generic support group as a potential control against the effect of time and attention on recovery from PTSD [42]. Earlier research has, however, suggested that the implementation of such alternative methods may be deemed insensitive and unfair by family members of children in the control group [44].

Second is the lack of a specific PTSD screen in the study, a shortcoming common to other research performed with the CD-RISC, which makes it difficult to place our findings in the context of previous research. Two earlier studies of the CD-RISC, which showed that treatment of PTSD significantly improves resilience parallel to symptom reduction [22,23], partially support our findings. However, because our study was limited to assessing resilience by request of local health authorities, who were concerned about negative impacts of trauma assessment, its results cannot help clarify to what extent low resilience scores might mirror PTSD pathology [45-47].

Third, although we have uncovered that traumatic exposure appears to modify the intra-subject change in resilience attributable to the intervention, we could not explore whether hostages and non-hostages at the same levels of trauma severity differed significantly in their resilience changes. With 94 study participants the analyses were not sufficiently powered to answer this question. Small and missing cell sizes of non-hostages at higher levels of trauma experiences precluded the type of doubly-stratified analysis--or 3-way interaction of time, hostage status, and losses or injuries--which would be necessary to draw such inferences.

Three additional points should be highlighted. One, the results should be read with some caution, as our analyses are sensitive to a type of regression-to-the-mean effect. That is, the baseline resilience reports of hostages may be an underestimate of the true value, owing to the extent of trauma experiences associated with the hostage taking. In this case, it would be natural that the reported resilience of this group would improve substantially over time, conceivably even in the absence of an intervention such as ours. Two, at present, there are no CD-RISC norms. We are thus left to consider the pre-/post-intervention changes in CD-RISC scores in a purely statistical manner, without a clear context for the clinical meaning of a given CD-RISC score or change. Efforts should be directed to independently establishing validity and reliability of the instrument for this age group, as well as developing age-related norms, so that future research of this kind may have greater clinical meaning. Finally, our study was not designed to identify mechanisms for resilience enhancement. In fact, we did not provide specific resilience training to the counselors and therapists; rather, we selected activities that we hypothesized would naturally lead to resilience enhancement, which renders impossible any isolation of mediating influences. Thus, the observed changes in resilience cannot be directly attributed to any single element of the intervention, and it remains possible that the occasional therapeutic intervention, provoked by daytime and evening activities (e.g., first aid training, game playing, music, art) explains our findings. Research has suggested that there are multiple pathways to resilience change, and individuals are remarkably resilient to extremely traumatic exposures [48,49]. Future controlled studies should therefore be systematized so that the relative influences of various intervention components can be ascertained.

Despite the significant limitations of this study, we do know that mass violence not only manifests in posttraumatic mental health problems, but also in disrupted human attachment systems [50]. Therefore newly empowered social systems on the peer level, in addition to those of family and extended non-family associations [51], should be considered as a major element of future psychosocial response for afflicted communities. The results of this study do support this notion, and further suggests that a mixed trauma intervention group experience (i.e., groups comprising those who experienced traumatic events and those who have not) may improve the likelihood of an effective outcome.

## Competing interests

This paper is a matter of mission-orientated research, financed partially by SDC, in order to monitor the quality of their summer camps and to adapt future programs, if necessary. Stefan Vetter, M.D. was a lecturer of psychopharmacology in a temporary faculty position at the Department of Medical Psychology at the North Ossetian State Medical Academy (NOSMA), Vladikavkaz, Russian Federation. Beyond these affiliations, the authors declare no other conflicts of interests exist.

## Authors' contributions

SV: selection of the Russians research psychologist, principal investigator, interpretation, writing and revising. ID: conception, designer, principal investigator, statistical analysis, interpretation. MM: investigator, interpretation. RRH: conception, designer, statistical analysis, interpretation, revision. WTG: conception, designer, statistical analysis, interpretation, revision. ZK: conception, designer, statistical analysis, interpretation, revision. All authors read and approved the final manuscript.
